# The Impact of Personalized Dietary Interventions on Symptom Severity and Dietary Habits in Female Patients with Affective Disorders: A Pilot Study

**DOI:** 10.3390/nu18183060

**Published:** 2026-09-19

**Authors:** Kamil Nikel, Michał Rafał Stojko, Joanna Smolarczyk, Magdalena Stojko, Celina Gibner, Łukasz Kunert, Magdalena Piegza

**Affiliations:** 1 Students’ Scientific Club, Department of Psychiatry, Faculty of Medical Sciences in Zabrze, Medical University of Silesia, 40-055 Katowice, Poland; stojko.michal.07@gmail.com; 2 Department of Psychiatry, Faculty of Medical Sciences in Zabrze, Medical University of Silesia, 40-055 Katowice, Poland; 3 Independent Researcher, 40-000 Katowice, Poland

**Keywords:** nutritional psychiatry, affective disorders, bipolar disorder, recurrent depression, gut–brain axis, dietary intervention, omega-3 fatty acids, sodium butyrate

## Abstract

Introduction: Rapid advances in nutritional psychiatry highlight the central role of the gut–brain axis in the pathophysiology of mood disorders. Despite strong theoretical evidence linking poor dietary habits and dysbiosis to increased subclinical inflammation, standardized nutritional assessments and personalized dietary care are rarely implemented in routine clinical practice for patients with affective disorders. Aim of the study: The objective was to perform a prospective evaluation of the impact of personalized dietary and educational interventions on the severity of depressive, anxiety, and manic symptoms, as well as on the modification of selected anthropometric parameters and behavioral dietary patterns in female patients with bipolar disorder (BD) and recurrent depression. Materials and methods: This pilot study included a cohort of *n* = 25 female patients (mean age: 31.32 ± 11.23 years). The participants underwent a 12-week personalized dietary modification and nutritional education program (focused on reducing pro-inflammatory foods, establishing meal regularity, and providing targeted supplementation with omega-3 fatty acids, vitamin D3, magnesium, and sodium butyrate). Mental health status was evaluated at two time points (Point I—baseline, Point II—follow-up) using the GAD-7, HADS, MADRS, and YMRS scales. Body mass index (BMI) and dietary habit profiles were monitored concurrently. Results: At the endpoint, a significant reduction in depressive symptom severity was observed on the MADRS (from 15.52 ± 12.01 to 13.20 ± 11.66 points; *p* < 0.001) and HADS (from 17.00 ± 7.58 to 14.88 ± 7.82 points; *p* = 0.004). GAD-7 scores decreased from 8.60 ± 4.75 to 7.60 ± 4.63 points (*p* = 0.065),whereas YMRS scores remained stable (3.80 vs. 3.56 points). Overall, 72.0% of patients (*n* = 18) reported improved dietary habits, including increased rates of optimal hydration (from 36.0% to 56.0%; *p* = 0.031) and the adoption of targeted omega-3 supplementation (from 12.0% to 36.0%; *p* = 0.031). A significant correlation was demonstrated between improved diet quality and the reduction in depressive symptoms (R = −0.42; *p* = 0.036). Conclusions: This pilot study demonstrates that personalized dietary care is a feasible and safe adjunctive intervention for female patients with affective disorders, providing preliminary evidence of associations with reductions in depressive and anxiety symptoms alongside favorable modifications in health-related behaviors. These findings inform the design of future large-scale randomized controlled trials.

## 1. Introduction

Affective disorders, encompassing major depressive disorder (MDD) and bipolar disorder (BD), are leading contributors to the global burden of disease (GBD) and disability worldwide [1]. These conditions are characterized by a chronic, recurrent course, high rates of somatic comorbidity, and a marked impairment in quality of life, generating a substantial socioeconomic burden [2]. Although tailored pharmacotherapy and psychotherapy remain the cornerstones of clinical management, a considerable proportion of patients (estimated at 30–40%) fail to achieve full and sustained symptom remission [3]. Consequently, current research is increasingly exploring non-pharmacological adjunctive strategies to improve the efficacy of standard therapeutic protocols.

Over the past decade, rapid advances in neurobiology have led to the emergence of nutritional psychiatry as a distinct discipline [4]. This field is grounded in robust empirical evidence demonstrating that diet quality directly influences the structure and function of the central nervous system. Epidemiological studies consistently link the Western dietary pattern (rich in ultra-processed foods, refined sugars, and trans fatty acids) to an elevated risk of onset and a more severe course of depressive episodes [5]. Conversely, dietary patterns with established anti-inflammatory properties, such as the Mediterranean diet, exert neuroprotective effects and may attenuate the severity of psychopathological symptoms [6].

The molecular mechanisms linking diet to mental health are multidirectional and converge on the gut–brain axis [7]. Poor dietary habits drive intestinal dysbiosis, characterized by quantitative and qualitative alterations in the microbiome composition. This process compromises intestinal barrier integrity (often referred to as “leaky gut”), facilitating the translocation of bacterial endotoxins (LPS) into the systemic circulation and triggering chronic, low-grade inflammation [8]. Pro-inflammatory cytokines (such as IL-6 and TNF-alpha) cross the blood–brain barrier, disrupting neurotransmitter metabolism (predominantly serotonin and dopamine) and downregulating brain-derived neurotrophic factor (BDNF) expression, which directly exacerbates depressive and anxiety symptoms [9]. Additionally, deficiencies in key essential nutrients—such as omega-3 polyunsaturated fatty acids, vitamin D3, magnesium, and short-chain fatty acids (SCFAs), including sodium butyrate—substantially exacerbate neuronal oxidative stress [10].

Despite strong theoretical foundations and the growing recognition of nutritional psychiatry, nutritional status and dietary habits are rarely systematically evaluated in routine psychiatric practice. Standardized screening tools, such as the Nutritional Risk Screening 2002 (NRS 2002), although widely used in somatic inpatient wards, are seldom utilized in psychiatric settings [11]. More importantly, the literature lacks prospective clinical trials evaluating the efficacy of individualized, supervised dietary interventions in patients with mood disorders.

The present pilot study was designed to address this gap in knowledge. The primary objective was to assess the feasibility, safety, and preliminary associations between personalized dietary consultations and modifications and the severity of depressive, anxiety, and manic symptoms (assessed via standardized psychometric scales: GAD-7, HADS, MADRS, and YMRS), as well as changes in selected anthropometric parameters (BMI) and dietary habits in female patients with affective disorders.

## 2. Materials and Methods

### 2.1. Study Design and Timeframe

This prospective, open-label pilot study was conducted as part of the “Studenckie Koła Naukowe Tworzą Innowacje” grant (no. SKN/SP/630911/2025). The dietary intervention was initially designed as a 12-week program. The end of the intervention period for participants was predefined as the completion of this 12-week dietary education module, at which point the follow-up data collection occurred. The study was based at the Psychiatric Outpatient Clinic of the Medical University of Silesia in Katowice. Patients were approached and referred for screening during routine clinical visits by their attending psychiatrists. Prior to the initiation of study procedures, formal project approval was obtained, and all participants provided written informed consent. The Bioethics Committee of the Medical University of Silesia in Katowice (decision dated 28 April 2025; ref. no. BNW/NWN/0052/KB) determined that the study did not constitute a medical experiment and therefore did not require formal bioethical review.

### 2.2. Study Population and Eligibility Criteria

The initial target sample size was set at N = 30, based on standard recommendations for pilot feasibility studies. The final study cohort characteristics and completion rates are detailed in the Section 3. Recruitment was conducted among patients presenting with depressive, anxiety, or manic symptoms in the course of bipolar disorder, recurrent depressive disorder, or depressive episodes.

**Inclusion criteria were:**

1. Female sex and age over 18 years.
2. A psychiatrist-confirmed diagnosis of bipolar disorder (BD), recurrent depressive disorder, or a depressive episode in the course of dysthymia.
3. A clinically stable condition enabling cooperation in an outpatient or intermediate care setting.

**Exclusion criteria included:**

1. Lack of patient consent to participate in the study or to modify current dietary habits.
2. Severe comorbid somatic disorders precluding the safe implementation of the dietary intervention.

### 2.3. Research and Psychometric Instruments

A diagnostic assessment battery encompassing psychiatric and nutritional domains was employed. Evaluations were conducted at two time points: baseline **(Point I—baseline assessment)** and post-intervention **(Point II—endpoint assessment).**

- Psychiatric scales (mental health assessment):
  ○ Patient self-report: Generalized Anxiety Disorder 7-item scale (GAD-7) and Hospital Anxiety and Depression Scale (HADS).
  ○ Clinician-rated (evaluated by a psychiatrist): Montgomery–Åsberg Depression Rating Scale (MADRS) and Young Mania Rating Scale (YMRS).
- Nutritional and anthropometric tools:
  ○ Assessment of malnutrition risk and nutritional status using standardized screening instruments: Mini Nutritional Assessment (MNA) and Nutritional Risk Screening 2002 (NRS 2002).
  ○ Anthropometric measurements: body weight (kg), height (cm), and calculated body mass index (BMI).
  ○ A custom dietary habits questionnaire evaluating the consumption frequency of specific food groups (vegetables, fruits, whole grains, omega-3 sources, processed foods, and sweets), meal regularity, emotional eating, fluid intake, and dietary supplementation. Participants also provided a subjective rating of their overall diet on a 1–10 scale, where 1 indicated extremely poor dietary habits, and 10 represented excellent adherence to optimal nutritional guidelines.

### 2.4. Dietary and Educational Intervention Protocol

During the baseline visit (Point I), experienced clinical dietitians, in collaboration with the research team, conducted a comprehensive dietary assessment for each participant. Based on this evaluation, individualized dietary recommendations were established, tailored to personal nutritional requirements, food preferences, allergies, and clinical status.

Throughout the study, patients participated in a targeted nutritional education program delivered via an initial one-on-one consultation (comprising a comprehensive dietary interview and the issuance of personalized recommendations), followed by a dedicated educational session with a clinical dietitian, focused on:

- Eliminating dietary patterns that negatively impact the course of affective illness (e.g., restricting refined sugars, trans-fatty acids, fast foods, and savory snacks).
- Increasing the intake of nutrients with anti-inflammatory and neuroprotective properties (vegetables, fruits, high-quality protein, and healthy fats rich in omega-3 fatty acids).
- Introducing and monitoring targeted supplementation (including omega-3 fatty acids, vitamin D3, magnesium, zinc, B-complex vitamins, and sodium butyrate). Supplementation was recommended based on individual baseline dietary deficits identified during the initial assessment, utilizing standard over-the-counter dosages.
- Supporting regular meal timing, adequate hydration, and reducing emotional eating behaviors.

Following the 12-week intervention period (Point II), patients underwent a repeat comprehensive psychiatric, nutritional, and anthropometric evaluation to determine intervention efficacy and dietary adherence. Feasibility was assessed by tracking study retention and intervention adherence rates, while safety was measured by monitoring for any adverse events, such as the potential induction of manic states. 

### 2.5. Statistical Analysis

Statistical analyses were conducted using Statistica software (version 13.3, StatSoft, Inc., Tulsa, OK, USA).

Quantitative (continuous) variables, including age, body mass index (BMI), and individual psychometric scale scores (GAD-7, HADS, MADRS, YMRS), are expressed as arithmetic means with standard deviations (M ± SD) and ranges (minimum–maximum). 

Qualitative (categorical) variables, such as educational level, place of residence, dietary habits, and supplementation profile, are presented as absolute frequencies (*n*) and percentages (%).

Prior to comparative testing, the normality of continuous variable distributions was verified using the Shapiro–Wilk test. Given the pilot nature of the study (*n* = 25) and the presence of non-normally distributed data, differences in continuous and ordinal parameters between baseline (Point I) and endpoint (Point II) were evaluated using the non-parametric Wilcoxon signed-rank test exclusively, maintaining consistency across all psychometric evaluations. Effect sizes for paired comparisons were calculated using Hedges’ g to account for the small sample size.

Paired nominal and dichotomous variables (e.g., changes in supplement use: yes/no, or meal regularity pre- vs. post-intervention) were compared using McNemar’s test. Associations between selected dietary habits and clinical improvement on psychometric scales were examined using Pearson’s chi-square (χ^2^) test of independence or Fisher’s exact test (in cases of low expected cell counts in contingency tables). Correlations between changes in subjective diet ratings and reductions in depressive and anxiety symptom severity were assessed using Spearman’s rank correlation coefficient (R).

For all statistical analyses, a two-tailed *p*-value of <0.05 was considered statistically significant.

## 3. Results

### 3.1. Demographic Characteristics, Baseline Nutritional Status, and Psychiatric Profile of the Study Cohort

Initially, 30 eligible patients were approached for participation; 3 declined consent to participate, and 2 did not complete the endpoint assessment, resulting in a final analyzed cohort of N = 25 women with affective disorders (Table 1). The cohort had a mean age of 31.32 ± 11.23 years (range: 19–66 years). Regarding educational attainment, 44.0% (*n* = 11) had completed higher education, 40.0% (*n* = 10) secondary education, and 16.0% (*n* = 4) vocational education. The majority of participants (56.0%, *n* = 14) resided in large cities with >200,000 residents. The mean duration of psychiatric treatment was 3.89 ± 4.58 years (median: 3.0 years). Analysis of the clinical profile revealed that somatic comorbidities were present in 28.0% of patients (*n* = 7; primarily thyroid disorders: 16.0% and PCOS: 8.0%), while additional psychiatric diagnoses were present in 24.0% (*n* = 6; including PTSD, personality disorders, ADHD, and anorexia nervosa).

At baseline (Point I), nutritional risk assessment using the NRS 2002 scale indicated that **48.0% of patients (*n* = 12) were at elevated nutritional risk (score ≥ 3)**. The baseline distribution of body mass index (BMI) categories was as follows: underweight (BMI < 18.5) was observed in 8.0% (*n* = 2), normal weight (BMI 18.5–24.9) in 48.0% (*n* = 12), overweight (BMI 25.0–29.9) in 16.0% (*n* = 4), and obesity (BMI ≥ 30.0) in 28.0% (*n* = 7).

### 3.2. Summary Analysis of Changes in Psychometric Parameters (Pre- vs. Post-Intervention)

**Figure 1:**
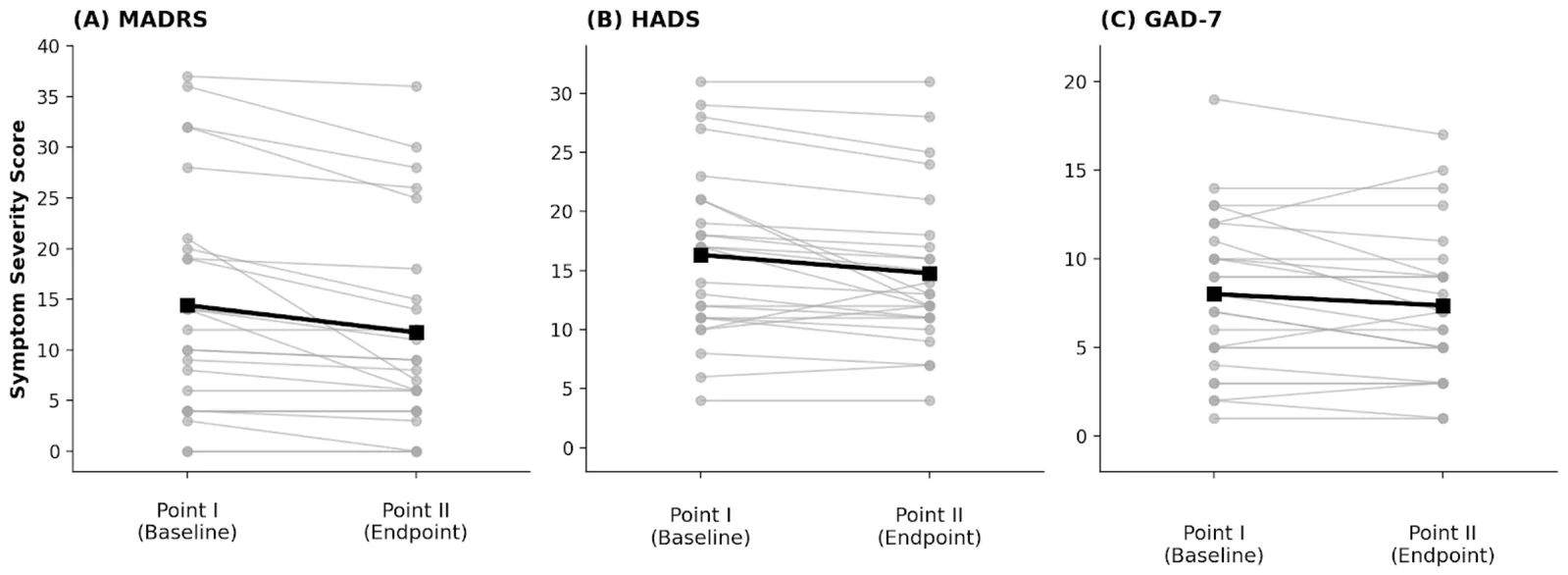
Paired dot plot visualizing individual patient pre- and post-intervention scores for (**A**) MADRS, (**B**) HADS, and (**C**) GAD-7, demonstrating the distribution of individual clinical trajectories across the cohort.

Figure 1 presents comparison of clinical outcomes before and after the 12-week dietary intervention demonstrated a comprehensive, marked reduction in symptom severity across all primary scales assessing anxiety and depressive symptoms.

Table 2 presents the complete statistical analysis of means and standard deviations for the entire cohort (*n* = 25).

At the endpoint (Point II), a highly statistically significant reduction in the severity of depressive symptoms was observed on the objective MADRS scale from 15.52 ± 12.01 (Me = 12.00) to 13.20 ± 11.66 points (Me = 9.00) (Z = 3.64; *p* < 0.001), with no worsening of scores observed in any of the study participants. Concurrently, a significant decrease in the total HADS score was demonstrated from 17.00 ± 7.58 to 14.88 ± 7.82 points (Z = 2.89; *p* = 0.004). Regarding generalized anxiety (GAD-7), a reduction in the mean score was observed from 8.60 ± 4.75 to 7.60 ± 4.63 points, representing a strong trend toward improvement (Z = 1.85; *p* = 0.065). Scores on the YMRS mania scale remained low and stable (3.80 ± 2.47 vs. 3.56 ± 2.50; Z = 2.00; *p* = 0.046). Although this minor reduction reached statistical significance, the effect size was negligible (g = 0.09) and lacks clinical relevance regarding affective symptom modification. Importantly, these results primarily served as a clinical assessment of safety, confirming the absence of hypomanic or manic state induction following the intervention. 

### 3.3. Statistical Analysis of Anthropometric Parameters (BMI)

Personalized dietary care was associated with exploratory trends in the metabolic optimization of patients’ body weight. Across the entire study cohort (*n* = 25), body weight and BMI remained stable, demonstrating no statistically significant differences (body weight: 73.48 ± 20.41 kg vs. 74.00 ± 20.08 kg, Z = 1.36; *p* = 0.174; BMI: 26.15 ± 6.70 kg/m^2^ vs. 26.26 ± 6.54 kg/m^2^, Z = 0.42; *p* = 0.674).

### 3.4. Exploratory Analysis of Behavioral Changes in Dietary Patterns and Habits

Given the modest sample size, detailed behavioral modifications and specific supplementation shifts are summarized below as exploratory findings without exhaustive formal hypothesis testing, minimizing the risk of false discovery. A comparison of response patterns from food frequency questionnaires revealed a shift in the health behaviors of the study participants (Table 3). Subjective assessment of diet on a 1–10 scale showed a statistically significant increase from a mean of 5.52 ± 2.08 (Me = 6.00) at Point I to 6.40 ± 2.20 (Me = 7.00) at Point II (Z = 2.82; *p* = 0.005). Although improvement in self-assessment was reported by a minority of patients (44.0%, *n* = 11), the magnitude of positive change within this subgroup was substantial enough to significantly shift the overall mean for the entire cohort.

Regarding hydration and dietary risk factors, a notable increase was observed at Point II in the proportion of patients consuming >6 glasses of fluids per day, rising from 36.0% (*n* = 9) to 56.0% (*n* = 14). Simultaneously, a clear reduction in exposure to dietary risk factors was observed, as the frequency of consuming fast food “daily” or “several times a week” was partially eliminated in favor of the “rarely” or “never” categories.

The intervention effectively raised patients’ awareness regarding the role of neurosupportive substances. At Point II, the prevalence of omega-3 fatty acid supplementation increased markedly (rising from 12.0% to 36.0%). A trend towards an increase was noted in the use of vitamin D3 from 64.0% to 84.0% (*p* = 0.063). Furthermore, an increase was observed from 24.0% (*n* = 6) to 36.0% (*n* = 9) of patients taking magnesium. Notably, at Point I, none of the patients used butyric acid, whereas at Point II, 20.0% of patients (*n* = 5) introduced sodium butyrate into regular supplementation, and 12.0% (*n* = 3) initiated targeted probiotics.

Analysis of emotional eating and behavioral habits showed that at baseline, 68.0% of patients (*n* = 17) confirmed that they regularly snacked in response to stress or affective states. Following completion of the educational program, this proportion decreased to 52.0% (*n* = 13). Regarding meal regularity, at Point I, only 40.0% of patients (*n* = 10) consumed meals on a regular basis. At Point II, following the implementation of personalized plans, this figure increased to 68.0% (*n* = 17).

### 3.5. Correlation Between Dietary Modification and the Direction of Clinical Changes

Spearman’s rank correlation analysis demonstrated a significant negative correlation between the increase in subjective dietary assessment (Δ diet score) and the change in symptom severity on the MADRS scale (ΔMADRS) (R = −0.42; *p* = 0.036), confirming that greater adherence to dietary recommendations was associated with a more pronounced reduction in depressive symptoms.

## 4. Discussion

The primary objective of this pilot study was to assess the feasibility, safety, and preliminary efficacy signals of individualized dietary and educational interventions in female patients with affective disorders (bipolar disorder, recurrent depression, and dysthymia). The obtained results provide promising preliminary evidence suggesting that personalized nutritional care is a feasible approach that may be associated with reductions in the severity of depressive and anxiety symptoms. The reduction in mean depression scale scores demonstrated at the endpoint (Point II) (a 2.32-point decrease on the MADRS) corresponds directly with a self-reported score reflecting adherence to dietary recommendations. These findings are consistent with a recent meta-analysis published in Annals of Internal Medicine (2025) [12], which confirms that targeted dietary interventions and caloric optimization yield measurable benefits in reducing anxiety and depressive symptoms. Furthermore, similar conclusions emerge from prospective studies on personalized medical nutrition therapy, demonstrating that individual tailoring of dietary plans in patients with low mood leads to a significant reduction in depression scale scores, correlating with shifts in microbiome composition [13].

A key element analyzed was the substantial shift in the profile of targeted supplementation and its potential impact on mood stabilization. At Point II, a three-fold increase in the prevalence of omega-3 fatty acid supplementation was recorded (up to 36.0%), along with a trend towards an increase in vitamin D3 supplementation (up to 84.0%). From a neurobiological perspective, omega-3 polyunsaturated fatty acids (particularly eicosapentaenoic acid—EPA) are integral components of neuronal cell membranes, influencing synaptic fluidity and neurotransmission. Importantly, both omega-3 fatty acids and vitamin D3 exhibit potent immunomodulatory properties, inhibiting the inflammatory cascade by reducing the expression of pro-inflammatory cytokines (IL-1, IL-6, and TNF-α) and dampening microglial activation. In light of the inflammatory hypothesis of affective disorders, we hypothesize, based on existing literature, that the implemented supplementation could be associated with a reduction in subclinical neuroinflammation in the central nervous system, which might manifest as improved psychometric outcomes among patients demonstrating high therapeutic engagement. This relationship is supported, among others, by a randomized controlled trial (RCT) conducted by Saunders et al. (2022) [14], in which an omega-3-rich diet (combined with omega-6 restriction) in patients with bipolar I and II disorder led to a significant reduction in mood variability and improved affective stabilization [14]. This effect is also widely emphasized in current dietary recommendations for patients with bipolar disorder, highlighting omega-3 fatty acids and vitamin D3 as crucial cofactors supporting mood stability and reducing the risk of episode recurrence [15].

A particularly compelling and novel aspect of the study was the initiation of sodium butyrate supplementation by 20.0% of patients at the endpoint. Sodium butyrate is the salt of a short-chain fatty acid (SCFA) that serves as the primary energy substrate for colonocytes. Its inclusion in the therapeutic regimen aligns directly with the gut–brain axis paradigm. SCFAs not only reinforce the intestinal epithelial barrier, preventing the translocation of bacterial endotoxins into the systemic circulation, but also possess the ability to inhibit histone deacetylases (HDACs), which in turn stimulates brain-derived neurotrophic factor (BDNF) expression in the hippocampus. Elevated BDNF levels are a critical determinant of neuroplasticity, whereas its deficits correlate with the severity of depressive episodes. Although these mechanisms were not directly assessed in the present study, based on previous literature, we hypothesize that the introduction of sodium butyrate and targeted probiotic therapy in the study cohort might support pharmacological treatment via biological stimulation of neurogenesis. This concept remains speculative and requires formal evaluation in larger studies. This concept finds robust support in a systematic review published in early 2026 summarizing the current evidence on the antidepressant properties of butyrate [16]. Reviewing both animal models and human trials, the authors highlighted the substantial anti-inflammatory, neuroplastic, and epigenetic potential of direct butyrate supplementation in alleviating depressive symptoms [16]. Our pilot study, in which 20.0% of patients adopted sodium butyrate, represents one of the initial clinical steps substantiating this promising relationship among female patients with affective disorders in real-world clinical practice.

In an exploratory analysis of our aggregated data regarding the relationship between patient behaviors and their psychiatric status, distinct clinical patterns emerged. Patients who demonstrated the greatest reductions in symptom severity on depression scales were concurrently those who implemented the most profound changes in their diet. At Point II, they fully transitioned to consuming vegetables “at every meal,” drank >6 glasses of fluids per day, and reported a subjective dietary self-assessment score of ≥7. Conversely, patients whose psychiatric scale scores remained unchanged (the plateau group) exhibited the lowest degree of habit modification, illustrating a potential relationship between the degree of adherence to recommendations and therapeutic outcomes. This phenomenon confirms an association between nutritional psychiatry interventions and psychological well-being. The inverse pattern was observed in patients exhibiting a plateau effect (no change in psychiatric scale scores), whose dietary modifications were minimal, emphasizing the pivotal role of patient motivation and the necessity of ongoing educational support. Furthermore, we observed a substantial improvement in meal regularity. It is highly plausible that establishing a daily meal routine confers a direct psychological benefit by bolstering daily structure, which in itself may promote mood stabilization independent of purely nutritional pathways.

The potential bidirectional, regulatory effect of the intervention on body mass index (BMI) also warrants particular attention. Conventional dietary approaches typically focus solely on weight reduction in overweight and obesity. In an exploratory observation of extreme BMI categories in our study, favorable regulatory trends were noted. In the subgroup with baseline underweight (*n* = 2), both participants gained weight, reaching the normal range, whereas in the class II obesity subgroup, weight reduction was observed in some patients. Due to the small sample size, these observations retain a descriptive character. Resolving underweight status in psychiatric patients is critical, as profound protein-energy deficits severely impair monoamine synthesis (serotonin, norepinephrine) and exacerbate symptoms of anhedonia and apathy.

It is also worth emphasizing the importance of somatic and psychiatric comorbidity in the context of dietary intervention efficacy. In the study group, 16.0% of patients presented with thyroid dysfunction and 8.0% with polycystic ovary syndrome (PCOS)—conditions that directly impact hormonal balance and metabolic rate. The bidirectional weight regulation effect observed in this study—namely, safe BMI reduction in the obesity subgroup alongside BMI normalization in underweight patients (including a patient with a history of anorexia nervosa)—indicates that personalized dietary care maintains metabolic efficacy regardless of underlying endocrine and psychiatric burdens.

### Limitations

Despite demonstrating significant positive trends, the interpretation of the findings must account for several methodological limitations. First, this was a pilot study with a small sample size of only 25 participants, consisting exclusively of women, which precludes generalizing the findings to the broader population of individuals with affective disorders, including male patients. Additionally, the study employed an open-label, pre–post design in which each patient served as her own control. The absence of a conventional control group without dietary support makes it difficult to definitively isolate the effect of the nutritional intervention itself from concurrent pharmacotherapy, psychotherapy, or the natural fluctuating course of affective disorders. Another limitation is the self-reported nature of the collected data—information regarding dietary habits relied on subjective questionnaire responses, which entails the risk of recall bias or social desirability bias.

## 5. Conclusions

1. Personalized dietary interventions conducted within integrated care demonstrate clear potential in reducing the severity of anxiety and depressive symptoms in female patients with affective disorders.
2. Nutritional education is associated with favorable short-term modifications of health health behaviors, manifested by improved hydration, meal regularity, and the adoption of targeted gut-supportive and neuroprotective supplementation (omega-3 fatty acids, vitamin D3, and sodium butyrate).
3. These preliminary findings support the potential clinical utility of considering standardized nutritional screening tools (MNA, NRS 2002) in psychiatric settings and underscore the necessity for further randomized controlled trials in larger populations.

## Figures and Tables

**Table 1:** Demographic, clinical, and anthropometric characteristics of the study cohort (*n* = 25).

Characteristic	Value/Distribution for *n* = 25
Age [years] (Mean/Range)	31.3 years (19–66 years)
Place of residence:	
— Large city (>200,000 residents)	56.0% (14/25)
— Mid-sized city (50,000–200,000 residents)	20.0% (5/25)
— Small town (<50,000 residents)	12.0% (3/25)
— Rural area	12.0% (3/25)
Educational level:	
— Higher education	44.0% (11/25)
— Secondary education	40.0% (10/25)
— Vocational education	16.0% (4/25)
Duration of psychiatric treatment [years]	Mean: 3.89 ± 4.58 (Median: 3.0; range: 0.17–23)
Somatic comorbidities (total)	28.0% (7/25)
— Thyroid disorders (hypothyroidism/hyperthyroidism)	16.0% (4/25)
— Polycystic ovary syndrome (PCOS)	8.0% (2/25)
— Cardiovascular and other disorders	4.0% (1/25)
Additional psychiatric diagnoses (total)	24.0% (6/25)
— PTSD/Personality disorders	8.0% (2/25)
— ADHD/Anxiety disorders/Sleep disorders	12.0% (3/25)
— Eating disorders (*Anorexia nervosa*)	4.0% (1/25)
Baseline BMI profile:	
— Underweight (<18.5 kg/m^2^)	8.0% (2/25)
— Normal weight (18.5–24.9 kg/m^2^)	48.0% (12/25)
— Overweight (25.0–29.9 kg/m^2^)	16.0% (4/25)
— Obesity (≥30.0 kg/m^2^)	28.0% (7/25)

**Table 2:** Summary of psychometric scale scores in the entire study cohort (*n* = 25). Effect size calculated as Hedges’ g_av_ (using pooled standard deviation for paired samples).

Psychometric Scale	Baseline Assessment (Point I)M ± SD [Me (Q1–Q3)]	Endpoint Assessment (Point II)M ± SD [Me (Q1–Q3)]	Mean Difference (Δ)	Wilcoxon’s Test (Z)	*p* Value	Effect Size (g)	Clinical Interpretation
MADRS	15.52 ± 12.01 [12.00 (4.00–20.00)]	13.20 ± 11.66 [9.00 (4.00–15.00)]	−2.32	Z = 3.64	*p* < 0.001	g = 0.19	Highly significant reduction in depression
HADS	17.00 ± 7.58 [17.00 (11.00–21.00)]	14.88 ± 7.82 [13.00 (11.00 −17.00)]	−2.12	Z = 2.89	*p* = 0.004	g = 0.27	Significant reduction in affective symptoms
GAD-7	8.60 ± 4.75 [8.00 (4.50–11.50)]	7.60 ± 4.63 [7.00 (5.00–9.50)]	−1.00	Z = 1.85	*p* = 0.065	g = 0.21	Trend toward anxiety reduction
YMRS	3.80 ± 2.47 [4.00 (2.00–6.00)]	3.56 ± 2.50 [3.00 (2.00–6.00)]	−0.24	Z = 2.00	*p* = 0.046	g = 0.09	No induction of manic states

**Table 3:** Changes in the consumption patterns of key food groups across the entire cohort (N = 25).

Food Groups and Habits	Frequency	Point I Percentage of Patients (*n*)	Point II Percentage of Patients (*n*)
Daily vegetable consumption	• At every meal • Daily • Several times a week	24.0% (*n* = 6) 64.0% (*n* = 16) 12.0% (*n* = 3)	36.0% (*n* = 9) 48.0% (*n* = 12) 16.0% (*n* = 4)
Consumption of sweets and confectionery	• At every meal • Several times a week • Rare/never	32.0% (*n* = 8) 36.0% (*n* = 9) 32.0% (*n* = 8)	16.0% (*n* = 4) 36.0% (*n* = 9) 48.0% (*n* = 12)
Daily fluid intake	• 1–3 glasses • 4–6 glasses • More than 6 glasses	24.0% (*n* = 6) 40.0% (*n* = 10) 36.0% (*n* = 9)	8.0% (*n* = 2) 36.0% (*n* = 9) 56.0% (*n* = 14)

## Data Availability

The original contributions presented in the study are included in the article; further inquiries can be directed to the corresponding author.
